# Effective L-Tyrosine Hydroxylation by Native and Immobilized Tyrosinase

**DOI:** 10.1371/journal.pone.0164213

**Published:** 2016-10-06

**Authors:** Małgorzata Cieńska, Karolina Labus, Marcin Lewańczuk, Tomasz Koźlecki, Jolanta Liesiene, Jolanta Bryjak

**Affiliations:** 1 Department of Bioorganic Chemistry, Wrocław University of Technology, Wrocław, Poland; 2 Department of Chemical Engineering, Wrocław University of Technology, Wrocław, Poland; 3 Department of Polymer Chemistry and Technology, Kaunas University of Technology, Kaunas, Lithuania; Wageningen Universiteit, NETHERLANDS

## Abstract

Hydroxylation of L-tyrosine to 3,4-dihydroxyphenylalanine (L-DOPA) by immobilized tyrosinase in the presence of ascorbic acid (AH_2_), which reduces DOPA-quinone to L-DOPA, is characterized by low reaction yields that are mainly caused by the suicide inactivation of tyrosinase by L-DOPA and AH_2_. The main aim of this work was to compare processes with native and immobilized tyrosinase to identify the conditions that limit suicide inactivation and produce substrate conversions to L-DOPA of above 50% using HPLC analysis. It was shown that immobilized tyrosinase does not suffer from partitioning and diffusion effects, allowing a direct comparison of the reactions performed with both forms of the enzyme. In typical processes, additional aeration was applied and boron ions to produce the L-DOPA and AH_2_ complex and hydroxylamine to close the cycle of enzyme active center transformations. It was shown that the commonly used pH 9 buffer increased enzyme stability, with concomitant reduced reactivity of 76%, and that under these conditions, the maximal substrate conversion was approximately 25 (native) to 30% (immobilized enzyme). To increase reaction yield, the pH of the reaction mixture was reduced to 8 and 7, producing L-DOPA yields of approximately 95% (native enzyme) and 70% (immobilized). A three-fold increase in the bound enzyme load achieved 95% conversion in two successive runs, but in the third one, tyrosinase lost its activity due to strong suicide inactivation caused by L-DOPA processing. In this case, the cost of the immobilized enzyme preparation is not overcome by its reuse over time, and native tyrosinase may be more economically feasible for a single use in L-DOPA production. The practical importance of the obtained results is that highly efficient hydroxylation of monophenols by tyrosinase can be obtained by selecting the proper reaction pH and is a compromise between complexation and enzyme reactivity.

## Introduction

Tyrosinases (polyphenol oxidase, PPO, EC 1.14.18.1) are copper-containing oxidoreductases that are found in a wide range of bacteria, fungi, plants, and animals, reflecting their physiological importance. In the presence of oxygen, the enzyme exhibits two catalytic activities: monophenolase activity, in which monophenols are hydroxylated to *o*-diphenols, and diphenolase activity, in which diphenols are oxidized to their corresponding *o*-quinones, which are spontaneously polymerized into colored pigments [1,2]. The monophenolic and diphenolic routes require reorganization of the active center during both substrate cycles, a so-called lag-phase, and suicide inactivation, making the overall mechanism complex and still not fully accepted (e.g., [2–4]). Briefly, the ability to bind oxygen and the valence of copper cations affect the presence of one of four types of tyrosinase active sites: *met* [Cu^2+^–OH^-^–Cu^2+^] (*met*-Tyr), *deoxy* [Cu^+^–Cu^+^] (*deoxy*-Tyr), *oxy* [Cu^2+^–O_2_ –Cu^2+^] (*oxy*-Tyr), or *deact* [Cu^2+^ Cu^0^] (*deact-*Tyr). In the absence of a substrate, the predominant form of the enzyme is *met*-Tyr. It binds monophenols in a non-productive mode, while diphenols are converted to their corresponding *o*-quinones, releasing the *deoxy-*form. Due to the high affinity of oxygen for *deoxy-*Tyr, it is readily converted to *oxy*-Tyr, which catalyzes the hydroxylation of monophenols and oxidation of diphenols. *Deact-*Tyr is formed from *oxy*-Tyr during suicide inactivation, which was first explained by Land et al. (2007) [5]. Briefly, this inactivation occurs when the diphenolic substrates are processed as monophenols, and in this route, one of the copper ions in the active site undergoes reductive elimination, causing irreversible inactivation of tyrosinase.

The ability of tyrosinase to transform a wide spectrum of substrates has directed researchers’ attention to practical applications, such as the crosslinking of polysaccharides and proteins, modification of polymeric surfaces [6–8], construction of biosensors [9,10], or treatment of wastewater [11–13]. Nevertheless, the most interesting processes are these connected with the hydroxylation of monophenolics to products with antioxidant activity (e.g., caffeic acid and 7,3’,4’-trihydroxyisoflavone) and with anti-microbial and anti-inflammatory properties (e.g., hydroxytyrosol) [14–18]. Special attention is paid to the production of L-3,4-dihydroxyphenylalanine (L-DOPA), which is used in the treatment of heart disorders and Parkinson's disease [19–25].

The main problems in the enzymatic hydroxylation of L-tyrosine to L-DOPA are related to the low *oxy*-Tyr content in enzyme preparations (10–15%) [26] and the need to disrupt a cascade of reactions leading to melanin production. The *oxy*-Tyr level increases with increasing reaction time because the diphenolic products reactivate the production of this form from *met*-Tyr, accelerating L-tyrosine hydroxylation. However, higher L-DOPA concentrations increase the probability of suicide inactivation [3–5]. The low reaction yield, which is caused by melanin production, can be overcome by the reduction of DOPA-quinone back to L-DOPA, which is commonly achieved by the addition of ascorbic acid (AH_2_) [20, 22–24, 27–28]. In many reports, equimolar amounts of AH_2_ and L-tyrosine are used [19,20,29,30]. However, this procedure does not achieve a satisfying product concentration because some L-DOPA molecules can be enzymatically converted to DOPA-quinone and then reduced to L-DOPA by AH_2_ several times. However, the excess AH_2_ is unfavorable because ascorbic acid is recognized by tyrosinase as (i) a diphenolic substrate (ascorbate oxidase) [31] and (ii) a strong inactivator of the enzyme [32]. These phenomena and low tyrosinase stability are believed responsible for the low reaction yields (1.9–53% L-DOPA) [19,20,23,24,29,33].

To improve the stability of tyrosinase, it is recommended that the enzyme is used in immobilized forms, among which attachment to water-insoluble carriers seems to be the most popular and effective method (extensively review by Duran et al. (2002) [34]). Moreover, immobilization improved tyrosinase stability and allowed the immobilized enzyme to be reused [34] or applied in continuous processes [20,28,35]. Curiously, aeration was used in only a few approaches [14,20,28,30,36]; however, Peñalver et al. (2002) [37] showed that the oxidation of L-tyrosine and L-DOPA consumed 1.5 O_2_ and 1 O_2_ per molecule, respectively.

Several attempts were proposed to overcome the limitations of the process. L-DOPA accumulation in the reactor was reduced in a continuous system using tyrosinase that was immobilized on a membrane surface [19,35]. However, the low residence time resulted in a low reaction yield (up to 10%). To avoid the problem of AH_2_ supplementation, electroenzymatic systems [21,33,38] with native tyrosinase or tyrosinase that was immobilized on a cathode were proposed and yielded 40–96% conversion, depending on the form of the enzyme, but with a strong limitation on the cathode size. An interesting approach to reduce diphenolics accumulation and inactivation by AH_2_ was presented by Marin-Zamora et al. [28], which is based on the known complexation of *o*-diphenols with borate ions [39]. The authors used several monophenols in 0.5 M borate buffer, pH 9, for the complexation of AH_2_ and diphenols, aeration, and hydroxylamine (HA) to reduce *met*-Tyr to *oxy*-Tyr in the apparent absence of products and they reached 88–97% yields with immobilized tyrosinase in a recirculated packed-bed reactor. However, there was no information on the operational stability of the enzyme, and pH 9 is somewhat unsuitable for the enzyme (pH_opt_ is 7).

The use of tyrosinase for L-DOPA production is undoubtedly an interesting approach; however, it is not clear to what extent enzyme immobilization is economically justified. Thus, we performed studies comparing native and immobilized tyrosinase to pinpoint their weaknesses and strengthen these forms in two reaction systems: (i) with AH_2_ supplementation and (ii) that used by Marin-Zamora et al. [28]. The experiments with immobilized tyrosinase were preceded by an evaluation of the need for aeration, but all internal and external diffusional constraints and the sorption of substrates/products on the carriers’ surface were first examined to exclude the process constraints that could influence the response of the immobilized enzyme. In all reaction runs, the O_2_ concentration and by-product formation were monitored, whereas the L-tyrosine, L-DOPA, and AH_2_ concentrations were analyzed by HPLC, the only reliable method [24]. Special attention was paid to the stability of both enzyme forms in the components of the reaction mixtures. Moreover, three consecutive batch processes were run to assess the extent of inactivation of the bound enzyme. In this study, tyrosinase immobilized on cellulose-based carriers was used because the preparation was thermostable [40] and stable up to pH 9 [41].

## Materials and Methods

### Materials

L-tyrosine, (S)-2-amino-3-(3,4-dihydroxyphenyl)propanoic acid (L-DOPA), hydroxylamine (HA), ascorbic acid (AH_2_), Lowry’s reagent, bovine serum albumin, and divinyl sulfone were purchased from Sigma–Aldrich (Germany). The other chemicals, all of which were of analytical or ACS grade, were obtained from Avantor Performance Materials (Poland).

### Tyrosinase Isolation, Immobilization and Activity Assay

Tyrosinase was extracted from mushrooms, purified using the procedure described by Zynek et al. [42], and stored in a freezer (working solution). Briefly, very young and fresh mushrooms from a local producer were homogenized in cold acetone (-20°C; 30 min) and the mixture was centrifuged (7000 rpm; 20 min). The separated pulp was suspended in the buffer and the remaining acetone was removed with a vacuum pump. After the centrifugation the supernatant was subjected to salting-out (30% saturation; 0–4°C; 30 min), centrifugation (10000 rpm; 20 min). The ammonium sulfate concentration in supernatant was set at 60%. The precipitated proteins, enriched with tyrosinase, were dissolved in buffer and stored in a freezer. The enzyme preparation was not subjected to further purification up to homogeneity to make a cost of enzyme economically suitable for bioprocesses. Due to the short purification procedure, the preparation can be composed of several isoenzymatic forms which presence in *A*. *bisporus* was reported by several authors (e.g. [43, 44]).

The enzyme was immobilized on a cellulose-based carrier (DEAE-Granocel) by covalent attachment via the hydroxyl groups of the carrier, which was activated with divinyl sulfone [41]. The enzyme-carrier preparation was stored at 4°C and washed with buffer several times before each experiment.

Enzyme activity was measured in the presence of 1 mM L-tyrosine in 0.1 M phosphate buffer pH 7.0 and at 30°C. The reaction was monitored spectrophotometrically in a HELIOSα spectrophotometer (Unicam, Mahwah, NJ, USA) at 475 nm and the linear absorbance dependence vs. time (after a lag-phase period). The molar extinction coefficient of dopachrome (3600; [45]) was used for calculating the activity. The enzyme activity unit (U) was defined as the amount of enzyme that causes dopachrome production by 1 μmole in 1 min under reaction conditions. The mean analytical error was less than ± 2.5%. The specific activity of the working solution was 0.334 U/mg protein. The protein concentration was determined using Lowry’s method (Sigma procedure) with bovine serum albumin as a standard. The mean analytical error was less than ±2.5%. The activity of the immobilized enzyme (0.834 U in 1 mL of freely sedimented carrier) was measured in a well-mixed thermostatted batch reactor, as described by Labus et al. [41]. The mean analytical error was less than ±4.7%.

### Sorption of L-Tyrosine and L-DOPA on the Carrier

Ten milliliters of 0.5 mM L-tyrosine or L-DOPA were incubated in a batch reactor (30°C; 20 rpm) with 0.5 mL of thermally inactivated immobilized enzyme. Samples were withdrawn at 0.5 h intervals, and the UV-VIS spectra (200–600 nm; spectrophotometer GBC Cintra 303) were measured until the absorbance stabilized (3 h). Then, the samples were returned to the reactor. The L-DOPA and L-tyrosine concentration was calculated from calibration curves (280 nm; R^2^ = 0.9987 and 0.9986 for L-tyrosine and L-DOPA, respectively).

### Effect of Mixing and Temperature on the Initial Reaction Rate

The effect of the mixing rate on the initial reaction rate was tested in the batch reactor (30°C; 24 mL of 1 mM L-tyrosine in 0.1 M phosphate buffer, pH 7.0, and 0.2 mL of the sedimented immobilized enzyme in 0.8 mL of buffer) with a mixing speed from 35 to 200 rpm. The progression of the reaction was measured in 1 min intervals and the activity was calculated from a linear slope. The influence of temperature on the reaction rate was tested in the range of 15–30°C at 120 rpm. The native enzyme was used as a control.

### Determination of L-tyrosine, L-DOPA and Ascorbic Acid Concentrations by HPLC

L-tyrosine, L-DOPA and AH_2_ concentrations were determined by HPLC (LC6A pump, Shimadzu; 7725i manual injector, Rheodyne; 250 × 4.6 mm Hypersil GOLD aQ column, ThermoFisher; 2085 UV detector operating at 280 nm, ECOM). Data were collected using a DDI A/D interface (Chromatech) and Chroma software. Samples, which were acidified to pH 2.5–3.0 with 85% H_3_PO_4_, were eluted with 0.15% (w/w) phosphoric acid at a flow rate of 1.5 mL^.^min^-1^. The calibration curves were constructed in triplicate (R^2^ = 0.994, 0.997 and 0.960 for L-tyrosine, L-DOPA and AH_2_, respectively). AH_2_ concentrations were roughly estimated at longer reaction times due to presence of several transition states of ascorbic acid. Each sample was analyzed in triplicate.

### Effect of Oxygen on the Progression of the Reaction

The effect of the oxygen concentration on the progression of the reaction was tested in two thermostatted (30°C) and well-mixed (120 rpm) batch reactors, both of which were equipped with luminescent dissolved oxygen electrodes (HQ40D device; HACH LANGE). One of the reactors was additionally equipped with an air-sparger connected to a peristaltic pump.

In one process, 60 mL of 1.0 mM L-tyrosine and 2 mM AH_2_ in buffer were preheated to 30°C, the electrodes were equilibrated in the reactor, and the air-sparger was turned on. The reaction was initiated by adding 5 mL of the preheated native enzyme (working solution diluted to match the activity of 0.5 mL of immobilized tyrosinase) or 5 mL of the buffer containing 0.5 mL of the immobilized preparation. Samples (100 μL) were withdrawn at certain time intervals (after separation of the solids), acidified, and then analyzed with HPLC. The initial reaction points were used to determine the initial reaction rate (r_initial_) from a linear slope. Simultaneously, 2 mL samples were removed from the reactor (centrifuged for the immobilized preparation) and analyzed spectrophotometrically at 475 nm. After analysis, the samples were returned to the reactor (with carrier). The mean analytical error was less than ±2.5% for HPLC, ±3.0% for oxygen, and ±3.7% for absorbance measurements.

### L-tyrosine Hydroxylation

**Fig 1:**
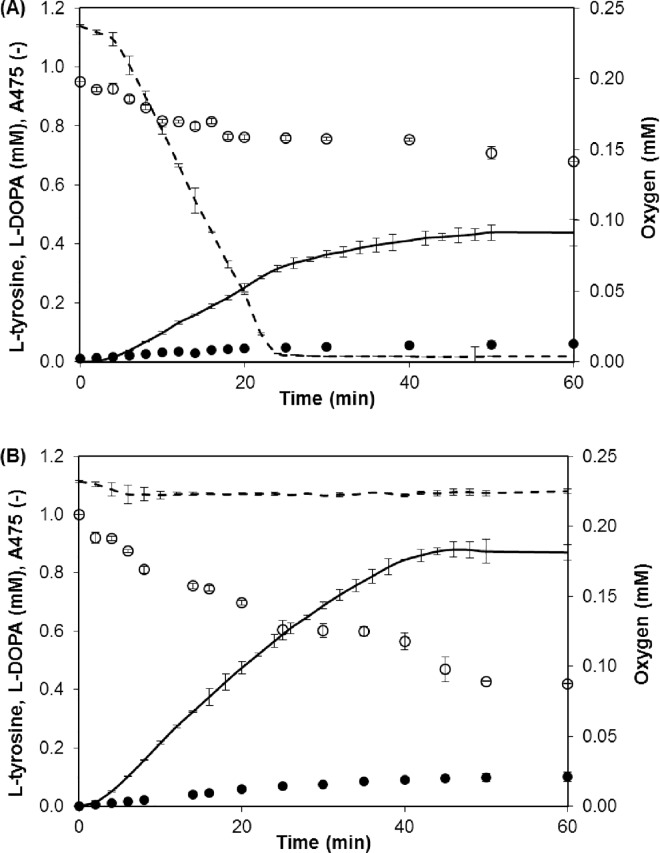
L-tyrosine Hydroxylation by Native Tyrosinase in a Batch Reactor. (A) Reaction without aeration of the reaction mixture. (B) Reaction with constant aeration. Symbols: oxygen (dashed line); A_475_ (solid line); L-tyrosine (○); L-DOPA (●). Reaction conditions: 1 mM L-tyrosine in 0.1 M phosphate buffer, pH 7; 30°C; and 20 rpm.

**Fig 2:**
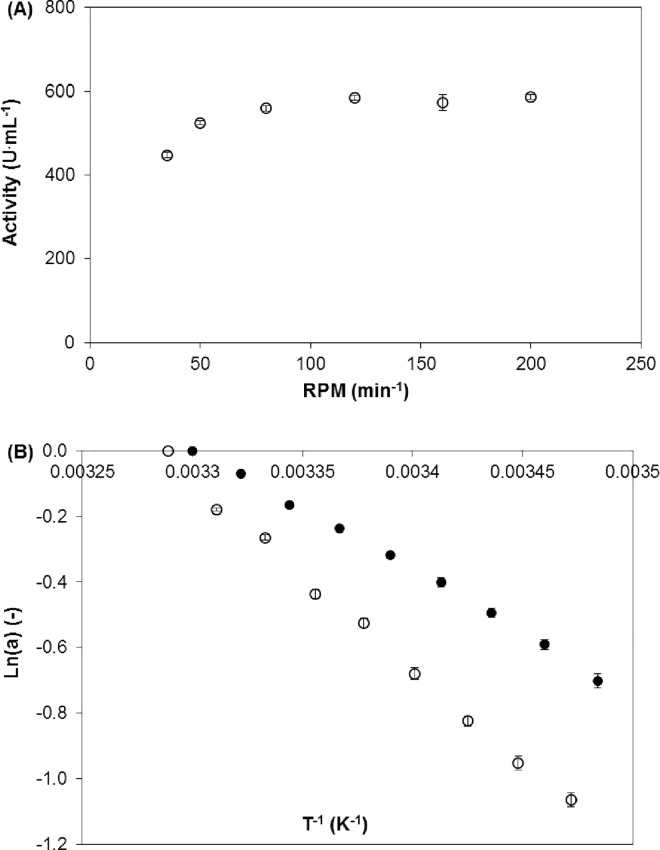
Effect of the Agitation Rate and Temperature on the Initial Reaction Rates (r). (A) Effect of the agitation rate using immobilized tyrosinase. (B) Arrhenius plots with native (black circles) and immobilized (empty circles) tyrosinase.

L-tyrosine hydroxylation reactions were performed with constant aeration and 1.0 mM L-tyrosine, as previously described. The processes differed with regard to the form of the enzyme (native or immobilized), the enzyme load (concentration), and the reaction mixture components: (i) 0.1 M phosphate buffer (PB), pH 7.0 or 0.5 M borate buffer (BB), pH 9, 8 or 7; (ii) 2.0 or 3 mM AH_2_; and (iii) 6.7 mM HA. Progression of the reaction was monitored for 1 h or until the L-DOPA concentration was no longer increased. The processes were performed at least in two independent runs. The standard deviations are plotted only in the figures shown in the main text. Other exemplary runs are presented in the Supporting Information S1 and S2 Figs and corresponding raw data are presented in S2 and S3 Appendixes for S1 Fig and S2 Fig, respectively. Raw data for Figs 1, 2, 4, 5, 7 and 8 are inserted in S1 Appendix.

### Tyrosinase Stability

Native tyrosinase was incubated in thermostatted batch reactors at 30°C in the presence of 2.0 mM AH_2_, 1 mM L-tyrosine, 1 mM L-DOPA or 6.7 mM HA in 0.1 M PB, pH 7.0, or in 0.5 M BB, pH 9.0. Samples were removed from the reactors after 1 hour and diluted 130-fold to minimize the influence of each component on the activity measurements (irreversible deactivation). In the reactions with the immobilized enzyme, the preparation was collected by filtration after 1 h of incubation, washed twice with buffer at 0.25 h intervals, and the activity was measured. Control experiments were performed in the appropriate buffers.

The operational stability of the immobilized enzyme was tested as described above. After 1 h of reaction, the immobilized enzyme was collected by filtration, washed twice with buffer, and re-suspended in fresh reaction mixture to begin the next cycle. Three independent experiments were performed and the results are reported as mean values and standard errors.

## Results and Discussion

### Impacts of Aeration and Diffusion Constraints on the Progression of the Reaction

Although L-tyrosine hydroxylation with immobilized tyrosinase has been the subject of many papers, some points remain to be clarified. First, the requirement for aeration should be clarified. For this purpose, L-tyrosine hydroxylation was performed in a well-mixed batch reactor with and without constant aeration (Fig 1). As shown in the figure, the lack of aeration resulted in a sudden decrease in the oxygen concentration and a reduced L-tyrosine conversion compared with the aerated mixture. The low level of L-DOPA in both conditions can be explained by its enzymatic oxidation to DOPA-quinone, which is converted to melanin pigments in series of non-enzymatic reactions. This process was confirmed by a gradual increase in absorbance at 475 nm (solid lines in Fig 1). To avoid any speculation that the enzyme can be inactivated by air bubbles, the stability of native and immobilized tyrosinase was tested in batch reactors with intensive aeration for 1 h. It was noted that the enzyme preserved 83 (native) and 100% (immobilized) of the initial activity, proving that the air bubbles had negligible influence on the termination of the reactions.

Processes with immobilized enzymes are heterogeneous reactions. The carrier used in this study may affect the enzyme’s properties by the sorption of substrates/products or by limiting diffusion. It was previously shown that the DEAE-Granocel carrier features wide pores, ensuring a very good microenvironment for tyrosinase activity [41]. As divinyl sulfone activation mostly affects the hydroxyl groups on the carrier’s surface, one can expect that the reaction should be kinetically controlled. To quantify the effects of the abovementioned limitations, three tests were performed, which are listed below.

1. The influence of agitation on the reaction rate was examined to determine the effect of external diffusion (Fig 2A). As shown in the figure, the external mass transport limitation could be eliminated by setting the agitation rate to no lower than 120 rpm, which minimizes the thickness of the solvent layer surrounding the carrier’s surface.
2. The influence of temperature on the initial reaction rate was evaluated to determine the effect of internal diffusion. This ‘temperature criterion’ is based on the hypothesis that higher temperatures affect the enzyme reaction rates more than the diffusion rates. The linearity of native (no diffusion limitations) and immobilized tyrosinase on the Arrhenius plot (Fig 2B) demonstrated a lack of internal mass constraints on the reaction with the immobilized enzyme in the range of tested temperatures.
3. The partitioning effect was evaluated by the sorption of L-tyrosine or L-DOPA on the carrier with thermally denatured tyrosinase. Constant concentrations of both compounds were observed in the bulk solution during 3 h of contact (0.500±0.008 mM for L-tyrosine and 0.500±0.010 mM for L-DOPA). Hence, an effect of the reactants’ sorption was excluded.

In summary, it was shown that aeration of the reaction mixture is required, particularly when the enzyme exhibited reasonably high activity. Moreover, tyrosinase immobilized onto DEAE-Granocel is characterized by a lack of partitioning effects and internal diffusion constraints, whereas the external diffusion constraints can be minimized by an effective mixing rate. These reaction conditions allow us to directly compare L-tyrosine hydroxylation using native and immobilized tyrosinase regarding their observed behaviors’.

### Enzymatic L-tyrosine Hydroxylation in Phosphate Buffer with Ascorbic Acid

**Fig 3:**
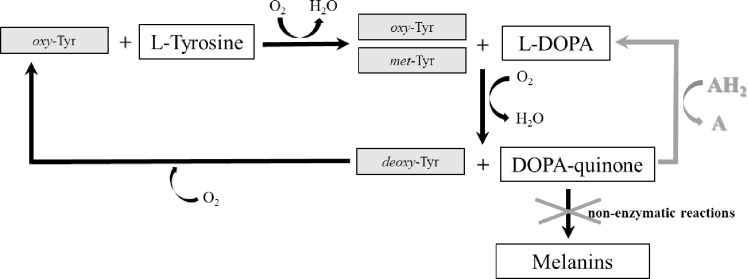
Simplified Schematic Representation of L-tyrosine Hydroxylation by Tyrosinase. Reactions without ascorbic acid—black arrows and a reaction system with ascorbic acid (AH_2_) supplementation—gray arrow. More detailed information about the actions of tyrosinase on L-tyrosine and L-DOPA is summarized in [2–4].

**Fig 4:**
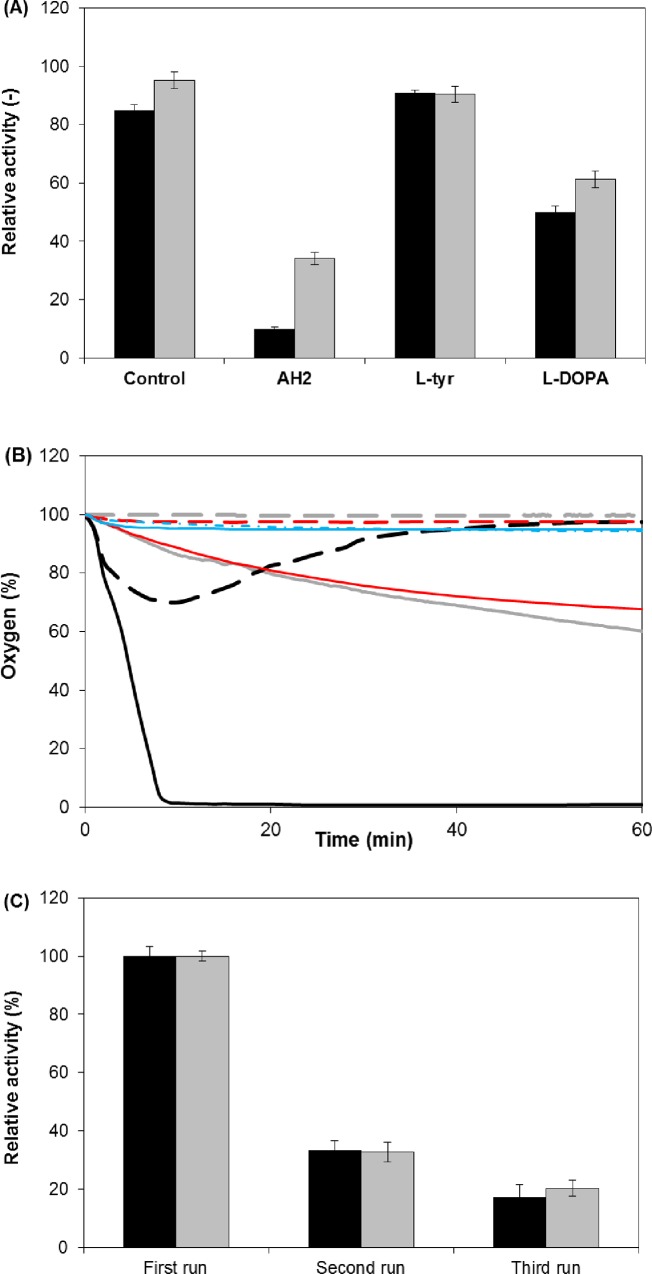
Stability of Tyrosinase in Reaction Mixture Components and Examples of Oxygen Consumption in Reaction Systems. (A) Relative activity of native (black) and immobilized tyrosinase (gray) after a 1 h incubation at 30°C in 0.1 M phosphate buffer, pH 7 (Control) containing 1 mM L-tyrosine (L-tyr), 1 mM L-DOPA, or 2 mM ascorbic acid (AH_2_). (B) An example of the effect of 1 mM L-tyrosine hydroxylation (gray lines) or 2 mM ascorbic acid oxidation (black lines) by native tyrosinase over time on the oxygen concentration in non-aerated (solid lines) and aerated (dashed or dotted lines) reaction mixtures. Red lines represents control experiments with 2 mM ascorbic acid whereas blue lines with 1 mM L-tyrosine, both without tyrosinase. Reaction conditions: 0.1 M phosphate buffer, pH 7; 30°C; and 20 rpm. (C) Relative initial reaction rates of immobilized tyrosinase in three consecutive 1 h processes in the batch reactor. Bars: black–measured from the increase in the L-DOPA concentration or white–measured from the decrease in the L-tyrosine concentration. Reaction conditions: 1 mM L-tyrosine and 2 mM ascorbic acid in 0.1 M phosphate buffer, pH 7; 0.133–0.235 O_2_; 30°C; and 120 rpm.

The most popular reaction system used to produce L-DOPA in the presence of ascorbic acid (AH_2_) by limiting melanin pigment formation is visualized in Fig 3. Due to planned use of 1 mM L-tyrosine in the tests, the AH_2_ concentration was adjusted to 2 mM to reduce the rapid depletion of ascorbic acid. However, the presence of the reactants had to be monitored to determine the extent of irreversible tyrosinase inactivation. Relative tyrosinase activity after a 1 h incubation in the presence of substrate, L-DOPA or AH_2_ is presented in Fig 4A, revealing that AH_2_ is a considerably stronger deactivator than L-DOPA, whereas the presence of L-tyrosine allowed tyrosinase to retain approximately 90% of its initial activity. It should be emphasized that noticeable O_2_ consumption was observed in the process with tyrosinase and AH_2_ and with lack of substrate and aeration (Fig 4B). Similar phenomenon was observed in the control without the enzyme. Assuming more intense spontaneous oxidation of AH_2_ in aerated mixture, large excess of ascorbic acid in relation to the substrate seems to be obvious.

**Fig 5:**
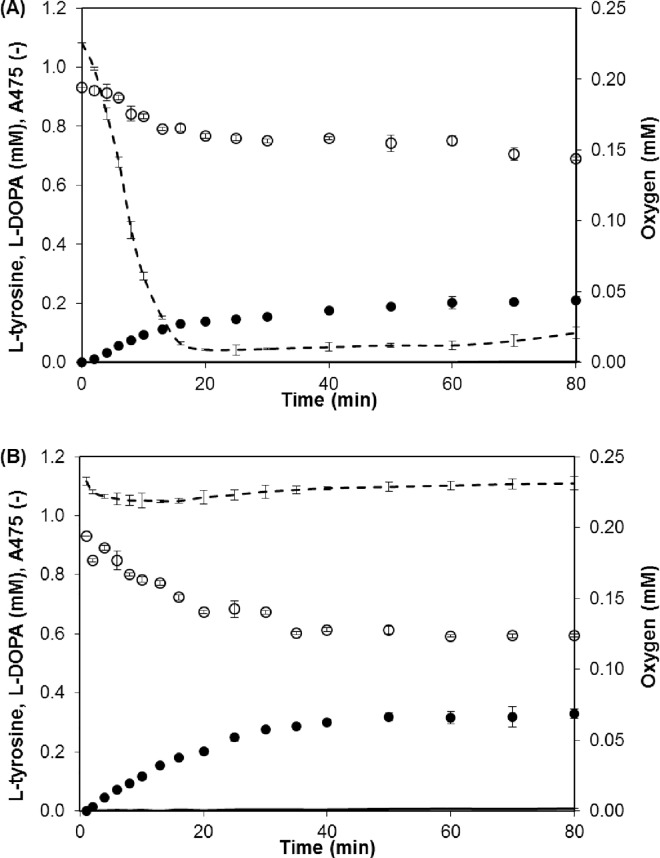
L-tyrosine Hydroxylation by Native Tyrosinase in a Batch Reactor. Reaction mixtures without (A) and with (B) aeration. Symbols: oxygen (dashed line); A_475_ (solid line); L-tyrosine (○); L-DOPA (●). Reaction conditions: 1 mM L-tyrosine and 2 mM ascorbic acid in 0.1 M phosphate buffer, pH 7; 30°C; and 20 rpm.

To better evaluate the effect of aeration, the same concentrations of native tyrosinase were applied in two parallel runs in the absence or presence of aeration (Fig 5). It can be observed that aeration produced higher substrate conversion but the dynamics of product growth decreased systematically and reached a plateau at approximately 80 min in both cases. The fact that the reaction was stopped in the aerated mixture agreed with an increase in the oxygen concentration up to the initial value, the high AH_2_ concentration (42 and 24% for the non-aerated and aerated mixtures, respectively), and low conversion for the substrate and L-DOPA. Generally, the experiment proved that tyrosinase stability was very low in the presence of all reaction components. For comparison, the presented processes can be characterized by a few useful parameters: (i) the initial reaction rates for L-tyrosine or L-DOPA; (ii) the amount of colored products produced, indicating AH_2_ depletion; (iii) the maximal conversion for L-tyrosine and L-DOPA; and (iv) the time required to achieve maximal conversion (Table 1, rows 1 and 2).

**Table 1:** Selected Parameters of Processes of 1 mM L-tyrosine Hydroxylation Using Native or Immobilized Tyrosinase in Phosphate Buffer (0.1 M, pH 7) in the Presence of Ascorbic Acid (AH_2_; 2 mM).

No.	Reaction system	r_L-tyrosine_[mM^.^min^-1^]	r_L-DOPA_[mM^.^min^-1^]	A_475_[–]	α_L-tyrosine_[%]	t_αmax_[min]	α_L-DOPA_[%]	t_αmax_[min]
Native enzyme								
1	Without aeration	-0.0116	0.0116	0.000	21.7*	60	21.1*	60
2	With aeration	-0.0120	0.0121	0.007	36.3	60	33.9	50
Immobilized enzyme								
3	Without aeration	-0.0081	0.0084	0.009	15.7*	60	16.3*	60
4	With aeration	-0.0105	0.0096	0.016	34.5*	60	30.6*	60
5	With aeration**	-0.0082	0.0080	0.000	33.6*	240	32.8*	240

The initial reaction rates (r) were calculated from the decrease in L-tyrosine concentrations or increase in L-DOPA concentrations; the level of colored products (A_475_); the maximal conversion for L-tyrosine hydroxylation to L-DOPA (α_L-tyrosine_; α_L-DOPA_), and the time required to achieve α_max_.

*- very low substrate/product change at the end of the reaction (enzyme still active)

**- 3 times with 2 mM AH_2._

Comparing the processes with and without aeration, as expected, higher initial reaction rates and conversions were noted for the system with an air-sparger, and effective DOPA-quinone conversion to L-DOPA was observed in both reactions (very low A_475_ values). The slightly shorter time required to terminate the reaction with the aerated mixture can be explained by the exposure of tyrosinase to higher L-DOPA concentrations.

Although the data from Fig 4A showed that immobilized tyrosinase was considerably more stable than the native enzyme, similar processes with this preparation (Table 1, rows 3 and 4; complete runs in Supporting Information S1 Fig) showed only slight differences: a higher level of colored products and lower conversion. We did our best to use the same number of activity units in the reactors with native and immobilized tyrosinase, but the initial reaction rates were slightly reduced compared with the native enzyme, which may be caused by the limited oxygen concentrations at the boundary layer of the carrier. In both processes (non-aerated and aerated), the reactions were not terminated, suggesting that tyrosinase stability was increased. Additionally, in the aerated reactor, an AH_2_ shortage was observed (higher A_475_); thus, in the next experiment, 3 portions of AH_2_ were added at 1 h intervals, and the reaction time was increased to 4 h (Table 2, row 5; S1 Fig), but without an evident improvement in conversion. Moreover, the preparation used for the reaction in row 4 of Table 1 was subjected to 3 successive runs of 1 h each, showing very poor stability of the bound enzyme in these processing conditions (Fig 4C).

**Table 2:** Selected Parameters of Processes of 1 mM L-tyrosine Hydroxylation Using Native or Immobilized Tyrosinase in Reaction Systems with Borate Buffer (BB; 0.5 M), and/or Ascorbic Acid (AH_2_; 2 mM), and/or Hydroxylamine (HA; 6.7 mM).

No.	Reaction system	r_L-tyrosine_[mM^.^min^-1^]	r_L-DOPA_[mM^.^min^-1^]	A_475_[–]	α_L-tyrosine_[%]	t_αmax_[min]	α_L-DOPA_[%]	tα_max_[min]
Native enzyme								
1	BB; pH 9; AH_2_	-0.0027	0.0024	0.001	7.0	50	2.5	60
2	BB; pH 9; HA	-0.0017	0.0013	0.039	13.2	60	5.3	60
3	BB; pH 9; AH_2_; HA	-0.0035	0.0030	0.000	17.4*	60	16.9*	60
4	BB; pH 9; AH_2_; HA	-0.0032	0.0027	0.010	25.1	360	24.5	300
5	BB; pH 8; AH_2_; HA	-0.0059	0.0058	0.007	97.8	360	95.4	360
6	BB; pH 7, AH_2_, HA	-0.0108	0.0099	0.028	98.3	240	96.8	240
7	BB; pH 7; AH_2_**; HA	-0.0123	0.0119	0.004	97.9	240	95.3	240
8	BB; pH 7; AH_2_**; HA	-0.0259^×2^	0.0246^×2^	0.121	99.6	150	93.5	120
Immobilized enzyme								
9	BB; pH 9; AH_2_; HA	-0.0030	0.0029	0.002	32.6*	480	31.6	300
10	BB; pH 8; AH_2_; HA	-0.0037	0.0036	0.011	76.5*	480	69.7	360
11	BB; pH 7, AH_2_, HA	-0.0045	0.0043	0.019	82.1*	480	70.0	240
12	BB; pH 7; AH_2_**; HA	-0.0131^×3^	0.0132^×3^	0.032	97.6	145	99.4	145

The initial reaction rates (r) were calculated from the decrease in L-tyrosine concentrations or increase in L-DOPA concentrations; the level of colored products (A_475_); the maximal conversion for L-tyrosine hydroxylation to L-DOPA (α_L-tyrosine_; α_L-DOPA_), and the time required to achieve α_max_.

* very low substrate/product change at the end of the reaction

** 3 mM AH_2_

^×2^ twice higher native enzyme load

^×3^ three-fold larger volume of immobilized enzyme

Based on the obtained results, one can conclude that the tyrosinase in the reaction system with AH_2_ is prone to strong inactivation by AH_2_ and L-DOPA. Even immobilization of the enzyme is not sufficient to reach considerably higher conversion, although increased stability can be observed in these processing conditions. The final result was that an average substrate conversion of 31–34% was obtained for aerated mixtures containing both enzyme preparations (other authors: 1.9–53%; [19,20,23,24,29,33]). Thus, efforts to decrease the effective concentrations of both reactants in the vicinity of the enzyme are necessary.

### L-DOPA production in boron buffer

**Fig 6:**
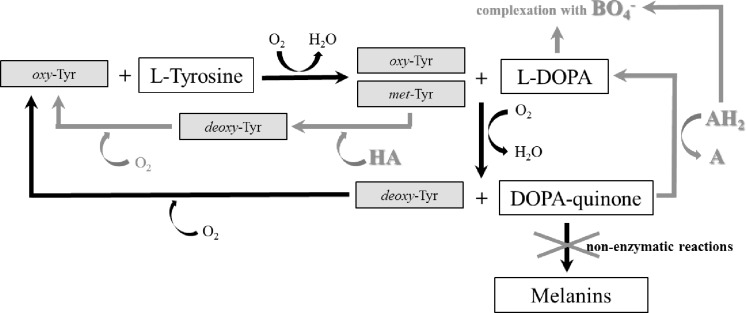
Simplified Schematic Representation of L-tyrosine Hydroxylation to L-DOPA by Tyrosinase in the Reaction System with Boron Buffer. Boron buffer was applied to create complexes with L-DOPA and ascorbic acid (AH_2_) that minimize suicide inactivation caused by both compounds; AH_2_ was added to reduce DOPA-quinone back to L-DOPA; hydroxylamine (HA) was added to reduce *met*-Tyr to *deoxy*-Tyr in reduced accessibility of L-DOPA and AH_2_. The scheme was designed according to that described by Marin-Zamora et al. [28]. The black arrows denote the reaction scheme without additives.

**Fig 7:**
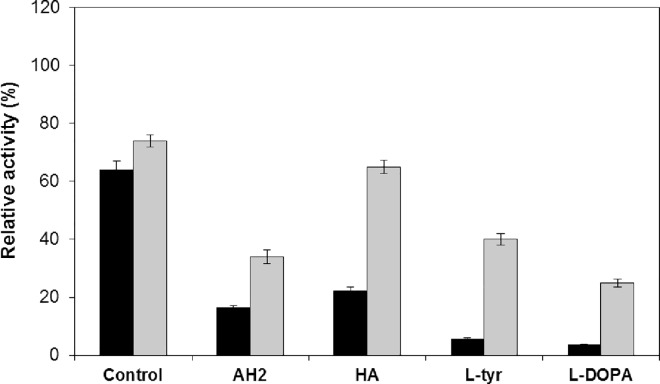
Stability of Tyrosinase in Presence of Reaction Mixture Components with Borate Buffer. Relative activity of native (black) and immobilized enzyme (gray) after 1 h incubation at 30°C in 0.5 M borate buffer, pH 9 (Control) containing 1 mM L-tyrosine (L-tyr), 1 mM L-DOPA, 2 mM ascorbic acid (AH_2_), or 6.7 mM hydroxylamine (HA).

The system in which L-DOPA and AH_2_ were complexed with borate ions was developed by Marin-Zamora et al. ([28] depicted in Fig 6) and applied to 4-*tert*-butyl-phenol, 4-methyl-phenol, 4-methoxy-phenol, *p*-hydroxyphenylpropionic acid and *p*-hydroxyphenylacetic acid hydroxylation with immobilized tyrosinase. The authors used 0.5 M boron buffer for the complexation of *o*-diphenols (1 mM) with AH_2_ (5 mM) and hydroxylamine (HA; 6.7 mM) to reduce *met*-Tyr to *oxy*-Tyr. It was expected that the reaction would be difficult because L-tyrosine and L-DOPA are physiological substrates for which the suicide inactivation mechanism has evolved, and because pH 9, which was selected for effective complexation, seemed somewhat unsuitable for this enzyme as its typical pH_opt_ is 7 (e.g. [41]). Studies of the stability of native and immobilized tyrosinase showed (Fig 7A) that the enzyme was more susceptible to irreversible inactivation in boron buffer (BB) than in PB. Increased activity loss was noted for L-tyrosine and AH_2_, although a concentration of 2 mM was applied. Additionally, it was not expected that HA would be a very strong deactivator. To assess the reaction rates, the processes were run using tested mixture components and the native enzyme (Table 2; rows 1–4; S2 Fig). First, a strong decrease in activity was noted (75%), which was higher than the values expected from our previous study (by 40% at pH 9; [41]) and could be assigned to a mixed effect of the pH and the high ionic strength of the BB buffer. Comparing the processes with AH_2_ or HA (Table 2; rows 1, 2), the need for both reagents is evident; HA alone caused the colored products to accumulate, whereas AH_2_ alone was responsible for very low conversion (low *oxy*-form regeneration). The presence of both reagents (Table 2; row 3) increased substrate conversion but did not complete the reaction within 1 h (S2 Fig). When the reaction time was prolonged to 6 h (Table 2, row 4), the conversion did not exceed 25%. It is obvious that the conversion could be increased by increasing the enzyme concentration, e.g., at least 4-fold. However, this solution to the problem seemed to be economically unfeasible. Therefore, we decided to change the pH of the reaction mixture to 8 and then to 7 to increase the reaction rates at the cost of complexation (Table 2; rows 5, 6; S2 Fig). The results showed that complete substrate conversion was achieved in both reactions with shorter reaction times at pH 7 and that the increased levels of colored products (weaker complexation) were reduced as the AH_2_ concentration increased from 2 mM to 3 mM (Table 2, row 7; S2 Fig). In the last experiment, to prove that the reaction runs are predictable, the enzyme concentration was increased two-fold (Table 2, row 8; S2 Fig), with an almost proportional increase in the initial reaction rates, a shorter reaction time and a concomitantly increased level of colored products. It could be assumed that the last effect can by reduced by increasing the AH_2_ concentration.

In summary, the present data show that full L-tyrosine hydroxylation can be achieved at pH 9, without the appearance of colored products, but it may be related to the at least 4-fold higher concentration of the protein used in the reaction. It was shown that full conversion can be achieved using the chosen enzyme concentration, but only at pH 8 or 7. The benefit of using pH 7 was the reduced reaction time, whereas the concentration of the colored products can be minimized by increasing the AH_2_ concentration. Interestingly, this reaction at the same pH in PB buffer terminated at 34% conversion, indicating that changes in buffer, ionic strength and HA additions increase substrate conversion through concerted action. It must be underlined that such high conversion was not previously noted in reactions with native tyrosine and were observed only when tyrosinase was immobilized on the cathode in the electrochemical system [21,38].

It was shown in Fig 7A that the immobilization of tyrosinase increased its stability in the reaction components, as shown in the process run at pH 9 for 6 h (Table 2, row 9; S2 Fig), in which conversion increased by 7% relative to the native enzyme (Table 1, row 4); the enzyme was still active toward L-tyrosine. However, the reaction was very slow and to increase the reaction rates, the pH was lowered to 8 and 7 (Table 2; rows 10, 11; S2 Fig). It was noted that as the pH decreased, the initial increase in the reaction rates with the immobilized enzyme was lower than in the reaction with the native enzyme, causing lower substrate conversion over time. This phenomenon may be associated with the slow rate of oxygen transport to the boundary layer of the carrier, suppressing enzyme activity. Surprisingly, although L-DOPA was still at the same maximal level in the reactions at pH 8, its concentration decreased after the maximum level was achieved at pH 7 (S2 Fig), both with a concomitant A_475_ increase and in the presence of AH_2_ in the reactor (28 and 38%, respectively). It can be attributed to the limited effective AH_2_ concentration triggered by complexation with the borate ions, even at these pH values.

**Fig 8:**
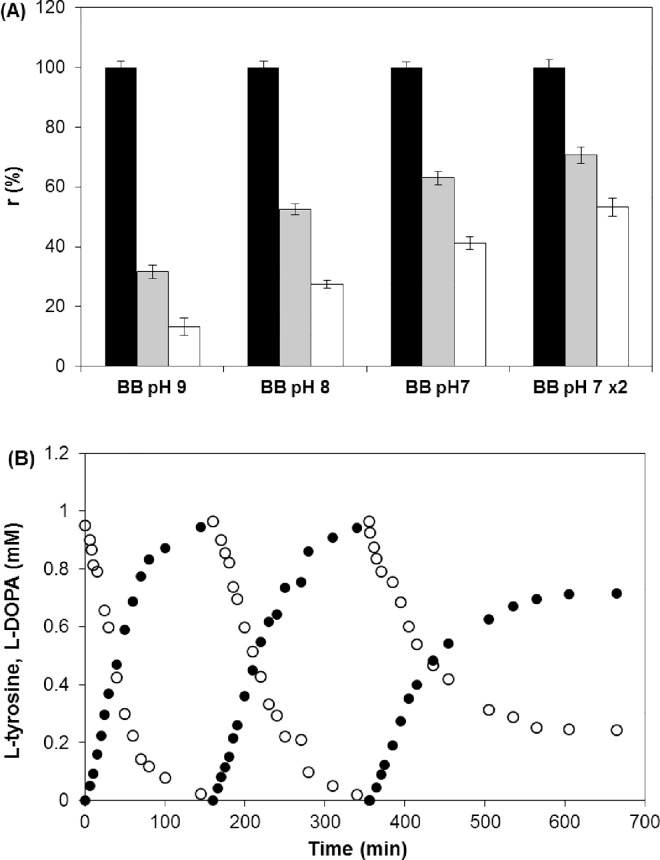
Operational Stability of Immobilized Tyrosinase. (A) Relative initial reaction rates of immobilized tyrosinase in three consecutive 1 h processes in the batch reactor. The black, gray, and white bars indicate the first, second, and third runs, respectively. (x2) indicates a two-fold higher enzyme load. (B) L-tyrosine hydroxylation by immobilized tyrosinase in three consecutive processes in the batch reactor. Symbols: L-tyrosine (○); L-DOPA (●). Reaction conditions: 1 mM L-tyrosine, 2 mM ascorbic acid, 6.7 mM hydroxylamine in 0.1 M boron buffer, pH 7; 0.130–0.239 O_2_; 30°C; and 120 rpm.

We conjectured that the merits of the higher stability of the immobilized enzyme could be counterbalanced by a slight increase in activity as the pH decreased and thus longer exposure of the enzyme to L-DOPA and AH_2_, due to weaker complexation. Contrasting results were obtained in three (1 h each) consecutive batch processes at pH 9, 8, and 7 (Fig 8A) because the initial reaction rates decreased after the first run as the pH decreased. At pH 7 and 8, the L-DOPA concentrations were considerably higher than at pH 9, but the activity was suppressed to a lesser extent. Thus, the inactivation noted at pH 9 may be attributed to unfavorable interactions of all of the compounds in the reaction mixture at this pH.

Generally, the data presented in Fig 8A clearly show that immobilized tyrosinase is unsuitable for L-DOPA production. It is obvious that very good results could be obtained with more enzyme (here 0.5 mL in 65 mL of reaction mixture with the upper limit as 50% of the carrier in a reactor volume [46]. Appropriate experiments in consecutive batches with an approximately two-fold larger volume of the carrier (r = 0.0083 mM/min for L-DOPA in the first run) showed better reactivity after the first and second runs (Fig 8A). However, the substrate conversion rate was increased by only 12% after 1 h compared with the first run in the reference reaction (23%), suggesting that full substrate conversion could not be achieved in this case. For that reason, three-fold larger volumes of the immobilized preparation were used for the next experiment (Table 2, row 12; S2 Fig), in which consecutive batches were added after the substrate had been depleted in the preceding reaction (Fig 8B). In this case, the increased contact of the enzyme with AH_2_ and L-DOPA was counterbalanced by its increased activity, and complete substrate conversion was achieved in two runs, but tyrosinase was inactivated in the third process. However, even a few runs with a higher enzyme concentration could not meet the economic criteria. Generally, the problem lies not in achieving an acceptable conversion but in the repeatability of consecutive runs, which depends on the reaction mechanism. Thus, we suggest that L-DOPA production by the native enzyme is more feasible than that with an expensive immobilized preparation.

## Conclusions

The conducted studies on the hydroxylation of L-tyrosine to L-DOPA have revealed several features of the use of native or immobilized tyrosinase. Initially, it was shown that immobilized tyrosinase does not suffer from adsorption of reactants or diffusion limitations. This allowed us to directly compare reaction runs with both forms of the enzyme that have comparable activities in batch reactors. Then, it was shown that aeration of reaction mixtures is required to maintain the efficiency of the process. It was also observed that a reduction in the concentrations of strong suicide inactivators in the tyrosinase microenvironment, namely L-DOPA and AH_2_, is profitable for increasing enzyme stability and can be realized by the use of boron buffer. The commonly applied pH 9 buffer produced satisfactory tyrosinase stability in the processing conditions but simultaneously reduced tyrosinase activity, particularly in reactions with the immobilized preparation. To solve this problem, we suggest that the pH of the reaction mixture should be reduced to pH 8 or 7 to ensure the full conversion of L-tyrosine to L-DOPA in the reactions with the native enzyme and 70% in the reactions with the immobilized preparation. A more than three-fold increase in the immobilized enzyme load achieved complete conversion in two successive runs, but in the third run, tyrosinase was inactivated due to processing of its main suicide inactivator, L-DOPA. In this case, the high cost of the immobilized enzyme preparation is not overcome by its repeated, long-term use; thus, immobilized tyrosinase should be used for the effective hydroxylation of many substrates (examples in [5]) with exception of all *o*-diphenols and triphenols that are suicide substrates [47]. However, the practical importance of the obtained results is the suggestion that very good efficiency for the hydroxylation of range of substrates by tyrosinase can be obtained by selecting the proper pH of the reaction mixture, which is a compromise between effective complexation and enzyme reactivity. Alternatively, in the case of L-DOPA production, inducible and pyridoxal 5'-phosphate dependent tyrosine-phenol lyase can be used. Lately, Koyanagi and coworkers [48] have showed that the recombinant *Erwinia herbicola* cells are capable to produce purer L-DOPA, lowering the cost of downstream processing in existing technology.

## Supporting Information

S1 Appendix Raw data for Figs 1,2,4,5,7, 8. (XLSX)

S2 Appendix Raw data for S1 Fig. (XLSX)

S3 Appendix Raw data for S2 Fig. (XLSX)

S1 Fig L-tyrosine hydroxylation by immobilized tyrosinase in a batch reactor with phosphate buffer. Data presented in S1 Fig correspond to data in Table 1, rows 3–5.
(DOCX)

S2 Fig L-tyrosine hydroxylation by native and immobilized tyrosinase in a batch reactor with borate buffer. Data presented in S2 Fig correspond to data in Table 2, rows 1–12.
(DOCX)
