# Supplementary material for: Effective L-Tyrosine Hydroxylation by Native and Immobilized Tyrosinase
Source: PLoS One. 2016 Oct 6;11(10):e0164213. doi: 10.1371/journal.pone.0164213 (PMC5053437; doi:10.1371/journal.pone.0164213)
Supplement: S1 Fig — Data presented in S1 Fig correspond to data in Table 1, rows 3–5. (DOCX) [file pone.0164213.s004.docx]

**Effective L-tyrosine Hydroxylation by Native and Immobilized Tyrosinase**

Małgorzata Cieńska^1^_,_ Karolina Labus^1^, Marcin Lewańczuk^1^, Tomasz Koźlecki^2^, Jolanta Liesiene^3^, Jolanta Bryjak^1*^

^1,2^ Faculty of Chemistry, Wrocław University of Technology, Wrocław, Poland

^3^ Faculty of Chemical Technology, Kaunas University of Technology, Kaunas, Lithuania

* Corresponding author; e-mail: [jolanta.bryjak@pwr.edu.pl](mailto:jolanta.bryjak@pwr.edu.pl)

**Supporting Information 1 (S1 Fig)**

**Data presented in S1_3 – S1_5 Figs correspond to data in Table 1, rows 3-5**

**
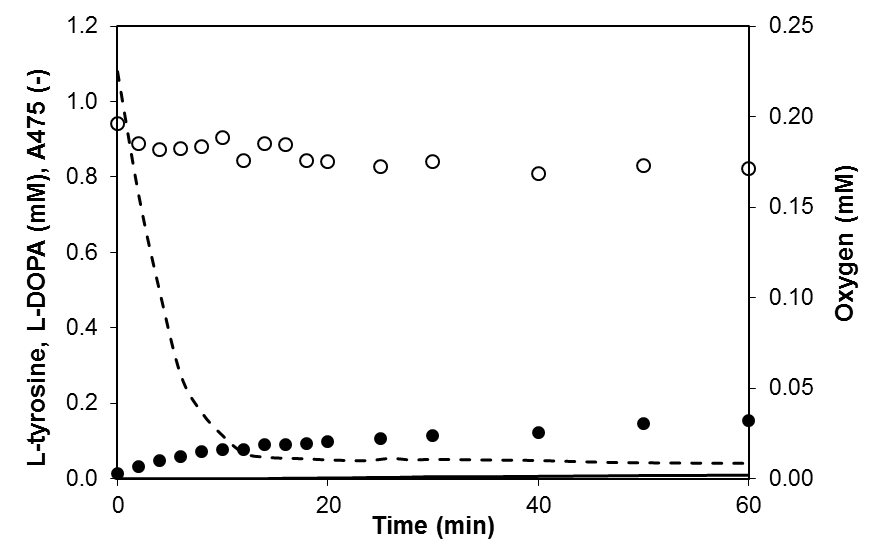
**

**Figure S1_3.** **L-tyrosine hydroxylation by immobilized tyrosinase in a batch reactor without aeration of reaction mixture.** Symbols: oxygen (dashed line); A_475_ (solid line); L-tyrosine (○); L-DOPA (●). Reaction conditions: 1 mM L-tyrosine and 2 mM ascorbic acid in 0.1 M phosphate buffer, pH 7; 30 °C; 120 rpm.

**
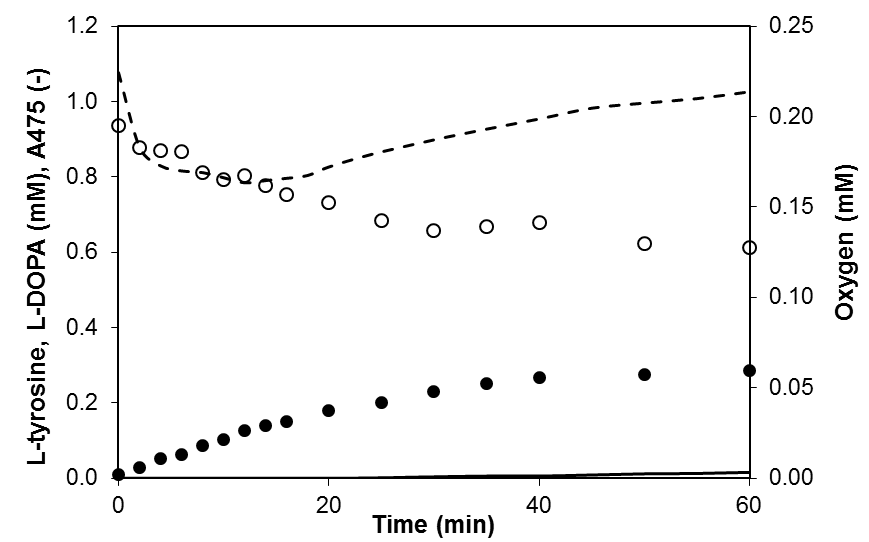
**

**Figure S1_4. As in Fig 1.3, apart from the presence of aeration.**

**
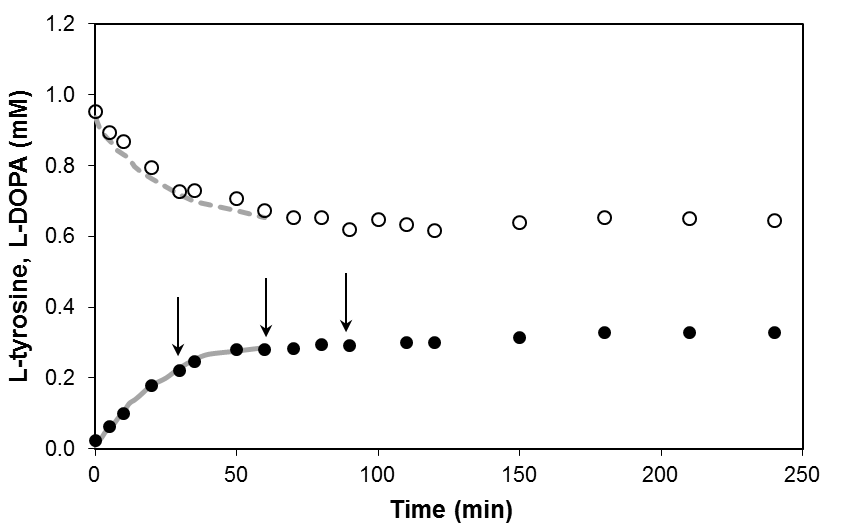
**

**Figure S1_5. As in Figure S1.4, apart from the addition of 3 portions of 2 mM AH_2_ (arrows).** Solid and dashed line – data from Fig. S1.4.
