# Supplementary material for: Effective L-Tyrosine Hydroxylation by Native and Immobilized Tyrosinase
Source: PLoS One. 2016 Oct 6;11(10):e0164213. doi: 10.1371/journal.pone.0164213 (PMC5053437; doi:10.1371/journal.pone.0164213)
Supplement: S2 Fig — Data presented in S2 Fig correspond to data in Table 2, rows 1–12. (DOCX) [file pone.0164213.s005.docx]

**Effective L-tyrosine Hydroxylation by Native and Immobilized Tyrosinase**

Małgorzata Cieńska^1^_,_ Karolina Labus^1^, Marcin Lewańczuk^1^, Tomasz Koźlecki^2^, Jolanta Liesiene^3^, Jolanta Bryjak^1*^

^1,2^ Faculty of Chemistry, Wrocław University of Technology, Wrocław, Poland

^3^ Faculty of Chemical Technology, Kaunas University of Technology, Kaunas, Lithuania

* Corresponding author; e-mail: [jolanta.bryjak@pwr.edu.pl](mailto:jolanta.bryjak@pwr.edu.pl)

**Supporting Information 2 (S2 Fig)**

**Data presented in Figs S2_1– S2.12 correspond to data in Table 2, rows 1-12**

**
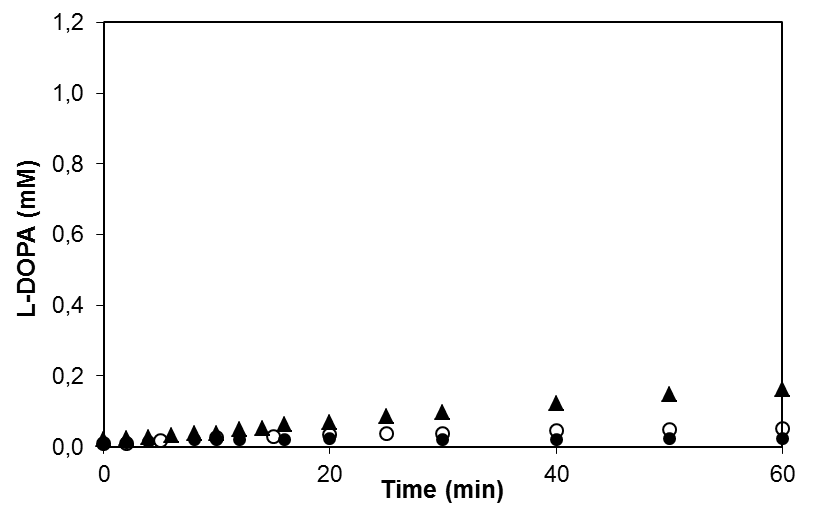
**

**Figure S2_1-3. L-tyrosine hydroxylation by native tyrosinase in a batch reactor with aeration of reaction mixture carried out in 0.5 M boron buffer, pH 9; 30 °C; 20 rpm in the presence of 2 mM AH_2_ (●); 6.7 mM HA (○) or 2 mM AH_2_ and 6.7 mM HA (▲).**

**
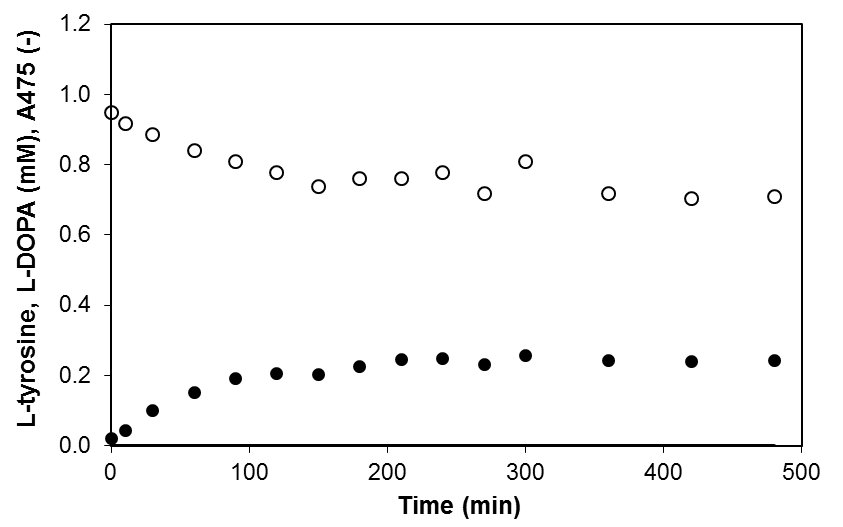
**

**Figure S2_4. L-tyrosine hydroxylation by native tyrosinase in a batch reactor with aeration of reaction mixture.** Symbols: A475 (solid line); L-tyrosine (○); L-DOPA (●). Reaction conditions: 1 mM L-tyrosine and 2 mM ascorbic acid in 0.5 M borate buffer, pH 9; 30 °C; 20 rpm.

**
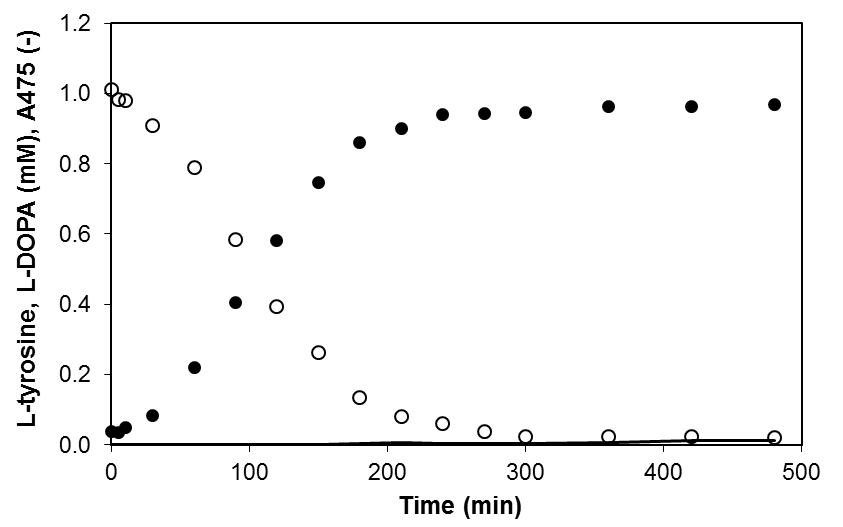
**

**Figure S2_5 s in Figure S2_4 but pH 8.**

**
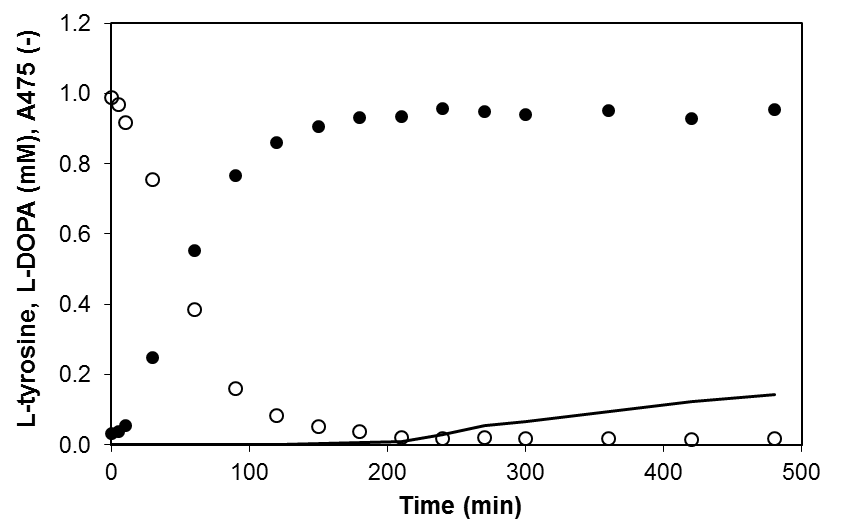
**

**Figure S2_6 as in Figure S2_4, but pH 7.0.**

**
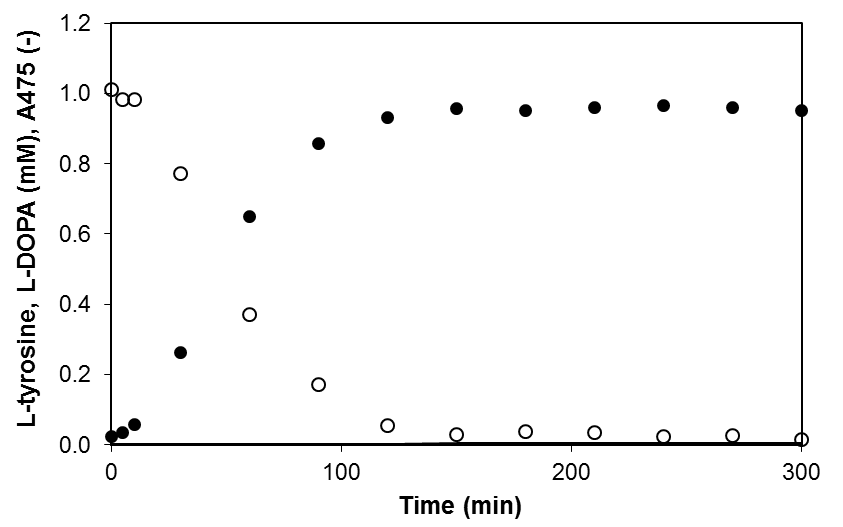
**

**Figure S2_7. As in Figure S2_6, but 3 mM AH_2_**


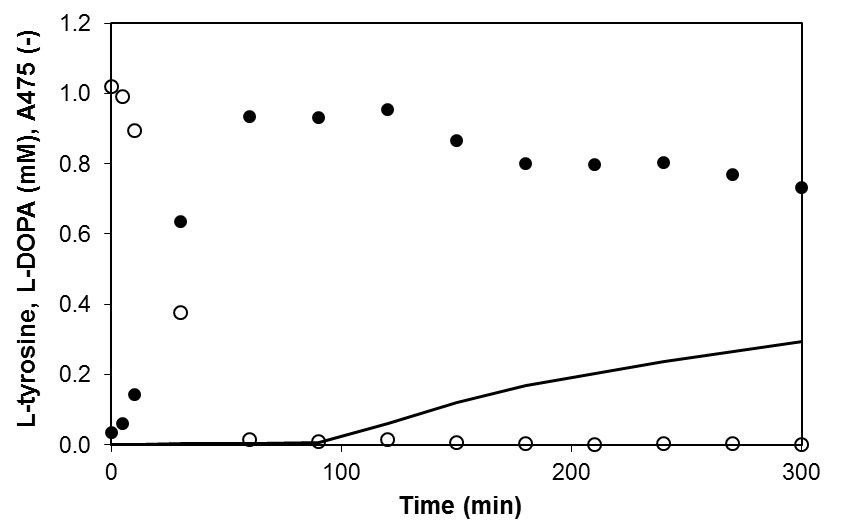


**Figure S2_8. As in Figure S2_6, but twice higher native enzyme load.**

**
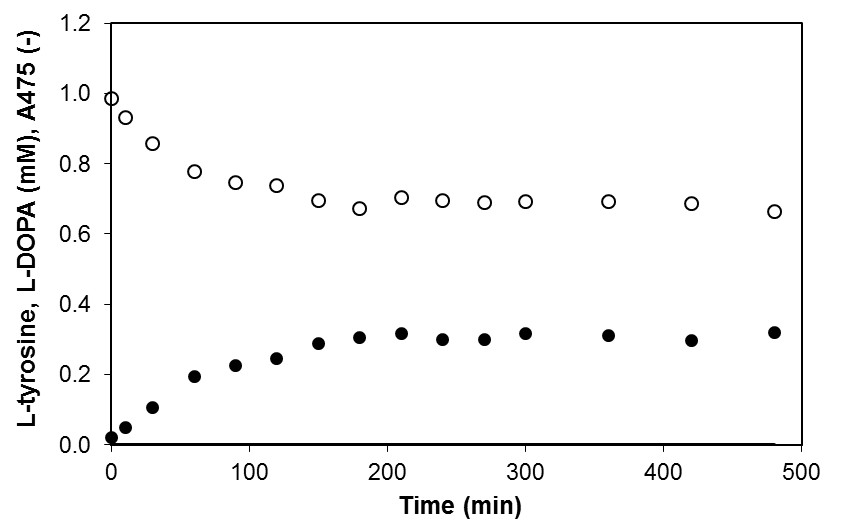
**

**Figure S2_9. L-tyrosine hydroxylation by immobilized tyrosinase in a batch reactor with aeration of reaction mixture.** Symbols: A475 (solid line); L-tyrosine (○); L-DOPA (●). Reaction conditions: 1 mM L-tyrosine and 2 mM ascorbic acid in 0.5 M borate buffer, pH 9; 30 °C; 120 rpm.

**
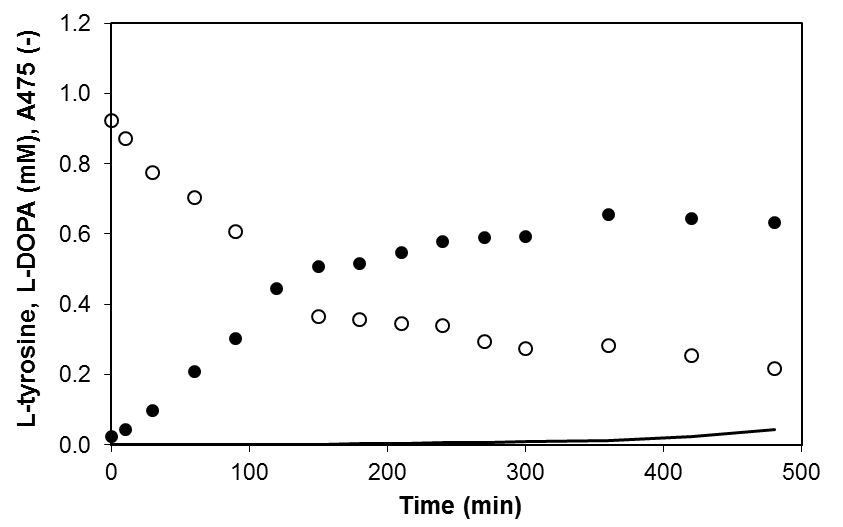
**

**Figure S2_10.** **As in Figure S2_9, but pH 8.0.**

**
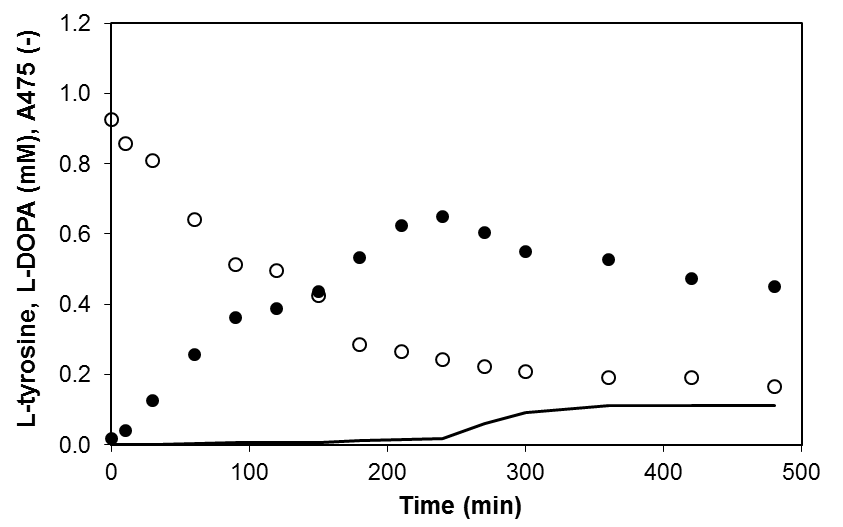
**

**Figure S2_11. As in Figure S2_9, but pH 7.0.**

**
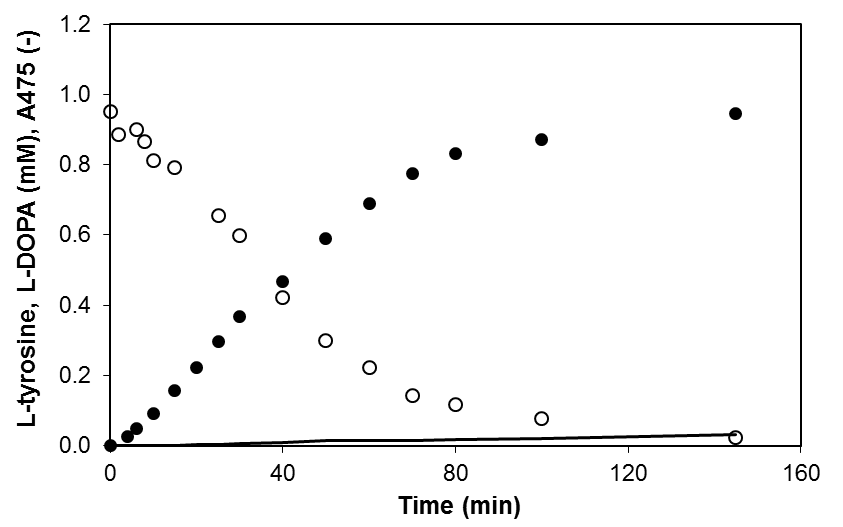
**

**Figure S2_12. As in Figure S2_11, but three-fold larger volume of immobilized enzyme and 3 mM AH_2_.**
